# A prospective prostate cancer screening programme for men with pathogenic variants in mismatch repair genes (IMPACT): initial results from an international prospective study

**DOI:** 10.1016/S1470-2045(21)00522-2

**Published:** 2021-11

**Authors:** Elizabeth K Bancroft, Elizabeth C Page, Mark N Brook, Sarah Thomas, Natalie Taylor, Jennifer Pope, Jana McHugh, Ann-Britt Jones, Questa Karlsson, Susan Merson, Kai Ren Ong, Jonathan Hoffman, Camilla Huber, Lovise Maehle, Eli Marie Grindedal, Astrid Stormorken, D Gareth Evans, Jeanette Rothwell, Fiona Lalloo, Angela F Brady, Marion Bartlett, Katie Snape, Helen Hanson, Paul James, Joanne McKinley, Lyon Mascarenhas, Sapna Syngal, Chinedu Ukaegbu, Lucy Side, Tessy Thomas, Julian Barwell, Manuel R Teixeira, Louise Izatt, Mohnish Suri, Finlay A Macrae, Nicola Poplawski, Rakefet Chen-Shtoyerman, Munaza Ahmed, Hannah Musgrave, Nicola Nicolai, Lynn Greenhalgh, Carole Brewer, Nicholas Pachter, Allan D Spigelman, Ashraf Azzabi, Brian T Helfand, Dorothy Halliday, Saundra Buys, Teresa Ramon y Cajal, Alan Donaldson, Kathleen A Cooney, Marion Harris, John McGrath, Rosemarie Davidson, Amy Taylor, Peter Cooke, Kathryn Myhill, Matthew Hogben, Neil K Aaronson, Audrey Ardern-Jones, Chris H Bangma, Elena Castro, David Dearnaley, Alexander Dias, Tim Dudderidge, Diana M Eccles, Kate Green, Jorunn Eyfjord, Alison Falconer, Christopher S Foster, Henrik Gronberg, Freddie C Hamdy, Oskar Johannsson, Vincent Khoo, Hans Lilja, Geoffrey J Lindeman, Jan Lubinski, Karol Axcrona, Christos Mikropoulos, Anita V Mitra, Clare Moynihan, Holly Ni Raghallaigh, Gad Rennert, Rebecca Collier, The IMPACT Study Collaborators, Lisa Adams, Julian Adlard, Rosa Alfonso, Saira Ali, Angela Andrew, Luís Araújo, Nazya Azam, Darran Ball, Queenstone Barker, Alon Basevitch, Barbara Benton, Cheryl Berlin, Nicola Bermingham, Leah Biller, Angela Bloss, Matilda Bradford, Nicola Bradshaw, Amy Branson, Charles Brendler, Maria Brennan, Barbara Bulman, Lucy Burgess, Declan Cahill, Alice Callard, Nuria Calvo Verges, Marta Cardoso, Vanda Carter, Mario Catanzaro, Anthony Chamberlain, Cyril Chapman, Michael Chong, Caroline Clark, Virginia Clowes, Lyn Cogley, Trevor Cole, Cecilia Compton, Tom Conner, Sandra Cookson, Philip Cornford, Philandra Costello, Laura Coulier, Michaela Davies, Christopher Dechet, Bianca DeSouza, Gemma Devlin, Fiona Douglas, Emma Douglas, Darshna Dudakia, Alexis Duncan, Natalie Ellery, Sarah Everest, Sue Freemantle, Mark Frydenberg, Debbie Fuller, Camila Gabriel, Madeline Gale, Lynda Garcia, Simona Gay, Elena Genova, Angela George, Demetra Georgiou, Alexandra Gisbert, Margaret Gleeson, Wayne Glover, Vincent Gnanapragasam, Sally Goff, David Goldgar, Nuno Gonçalves, Selina Goodman, Jennifer Gorrie, Hannah Gott, Anna Grant, Catherine Gray, Julie Griffiths, Karin Gupwell, Jana Gurasashvili, Eldbjørg Hanslien, Sigurdis Haraldsdottir, Rachel Hart, Catherine Hartigan, Lara Hawkes, Tricia Heaton, Alex Henderson, Rui Henrique, Kathrine Hilario, Kathryn Hill, Peter Hulick, Clare Hunt, Melanie Hutchings, Rita Ibitoye, Thomas Inglehearn, Joanna Ireland, Farah Islam, Siti Ismail, Chris Jacobs, Denzil James, Sharon Jenkins, Irene Jobson, Anne Johnstone, Oliver Jones, Sagi Josefsberg Ben-Yehoshua, Beckie Kaemba, Karen Kaul, Zoe Kemp, Netty Kinsella, Margaret Klehm, Roger Kockelbergh, Kelly Kohut, Monika Kosicka-Slawinska, Anjana Kulkarni, Pardeep Kumar, Jimmy Lam, Mandy LeButt, Dan Leibovici, Ramona Lim, Lauren Limb, Claire Lomas, Mark Longmuir, Consol López, Tiziana Magnani, Sofia Maia, Jessica Maiden, Alison Male, Merrie Manalo, Phoebe Martin, Donna McBride, Michael McGuire, Romayne McMahon, Claire McNally, Terri McVeigh, Ehud Melzer, Mark Mencias, Catherine Mercer, Gillian Mitchell, Josefina Mora, Catherine Morton, Cathryn Moss, Morgan Murphy, Declan Murphy, Shumi Mzazi, Maria Nadolski, Anna Newlin, Pedro Nogueira, Rachael O'Keefe, Karen O'Toole, Shona O'Connell, Chris Ogden, Linda Okoth, Jorge Oliveira, Edgar Paez, Joan Palou, Linda Park, Nafisa Patel, João Paulo Souto, Allison Pearce, Ana Peixoto, Kimberley Perez, Lara Petelin, Gabriella Pichert, Charlotte Poile, Alison Potter, Nadia Preitner, Helen Purnell, Ellen Quinn, Paolo Radice, Brigette Rankin, Katie Rees, Caroline Renton, Kate Richardson, Peter Risby, Jason Rogers, Maggie Ruderman, April Ruiz, Anaar Sajoo, Natale Salvatore, Victoria Sands, Francesco Sanguedolce, Ayisha Sattar, Kathryn Saunders, Lyn Schofield, Rodney Scott, Anne Searle, Ravinder Sehra, Christina Selkirk, Kylie Shackleton, Sue Shanley, Adam Shaw, Daniel Shevrin, Hannah Shipman, Zahirah Sidat, Kas Siguake, Kate Simon, Courtney Smyth, Lesley Snadden, Nita Solanky, Joyce Solomons, Margherita Sorrentino, Barbara Stayner, Robert Stephenson, Elena Stoffel, Maggie Thomas, Alan Thompson, Lizzie Tidey, Marc Tischkowitz, Audrey Torokwa, Sharron Townshend, Katy Treherne, Karen Tricker, Quoc-Dien Trinh, Vishakha Tripathi, Clare Turnbull, Riccardo Valdagni, Nicholas Van As, Vickie Venne, Lizzie Verdon, Marco Vitellaro, Kristen Vogel, Lisa Walker, Amy Watford, Cathy Watt, Ilana Weintroub, Shelly Weiss, Scott Weissman, Michelle Weston, Jennifer Wiggins, Gillian Wise, Christopher Woodhouse, Pembe Yesildag, Alice Youngs, Matthew Yurgelun, Fabiana Zollo, Judith Offman, Zsofia Kote-Jarai, Rosalind A Eeles

**Affiliations:** a Oncogenetics Team, Institute of Cancer Research, London, UK; b Cancer Genetics Unit & Academic Urology Unit, Royal Marsden NHS Foundation Trust, London, UK; c Clinical Genetics Unit, Birmingham Women's Hospital, Birmingham, UK; d Department of Medical Genetics, Oslo University Hospital, Oslo, Norway; e Genomic Medicine, Division of Evolution and Genomic Sciences, University of Manchester, Manchester Academic Health Sciences Centre, Manchester University NHS Foundation Trust, Manchester, UK; f North West Thames Regional Genetics Service, London North West University Healthcare NHS Trust, Harrow, UK; g St George's Hospital, Tooting, London, UK; h Parkville Familial Cancer Centre, Peter MacCallum Cancer Centre, Melbourne, VIC, Australia; i The Sir Peter MacCallum Department of Oncology, The University of Melbourne, Parkville, VIC, Australia; j Department of Medicine, The University of Melbourne, Parkville, VIC, Australia; k Division of Population Sciences, Dana Farber Cancer Institute, Boston, MA, USA; l Brigham and Women's Hospital, Boston, MA, USA; m University Hospital Southampton, Southampton, UK; n Wessex Clinical Genetics Service, Princess Anne Hospital, Southampton, UK; o Department of Genetics, University of Leicester, Leicester, UK; p University Hospitals Leicester, Leicester, UK; q Genetics Department and Research Center, Portuguese Oncology Institute (IPO Porto), Porto, Portugal; r Biomedical Sciences Institute (ICBAS), Porto University, Porto, Portugal; s Clinical Genetics Service, Guy's and St Thomas' NHS Foundation Trust, London, UK; t Clinical Genetics Service, Nottingham University Hospitals NHS Trust, Nottingham, UK; u Parkville Familial Cancer Centre, The Royal Melbourne Hospital, Parkville, VIC, Australia; v Colorectal Medicine and Genetics, The Royal Melbourne Hospital, Parkville, VIC, Australia; w Adult Genetics Unit, Royal Adelaide Hospital, Adelaide, SA, Australia; x Adelaide Medical School, University of Adelaide, Adelaide, SA, Australia; y The Genetic Institute, Kaplan Medical Center, Rehovot, Israel; z Biology Department, Ariel University, Ariel, Israel; aa North East Thames Regional Genetics Service, Institute of Child Health, London, UK; ab Yorkshire Regional Genetics Service, Leeds Teaching Hospitals NHS Trust, Leeds, UK; ac Fondazione IRCCS Istituto Nazionale dei Tumori, Milano, Italy; ad Clinical Genetics Service, Liverpool Women's Hospital, Liverpool, UK; ae Peninsular Genetics, Derriford Hospital, Plymouth, UK; af Royal Devon and Exeter Hospital, Exeter, UK; ag Genetic Services of Western Australia, King Edward Memorial Hospital, Subiaco, WA, Australia; ah Department of Paediatrics, University of Western Australia, Perth, WA, Australia; ai Hunter Family Cancer Service, Waratah, NSW, Australia; aj University of New South Wales, St Vincent's Clinical School, NSW, Australia; ak Cancer Genetics Clinic, The Kinghorn Cancer Centre, St Vincent's Hospital, Sydney, NSW, Australia; al Northern Genetics Service, Newcastle upon Tyne Hospitals NHS Foundation Trust, Newcastle upon Tyne, UK; am John and Carol Walter Center for Urological Health, Division of Urology, NorthShore University HealthSystem, Evanston, IL, USA; an Oxford Centre for Genomic Medicine, Oxford University Hospitals NHS Trust, Oxford, UK; ao Huntsman Cancer Institute, University of Utah, Salt Lake City, UT, USA; ap Hospital de Sant Pau, Barcelona, Spain; aq St Michael's Hospital, Bristol, UK; ar Duke Cancer Institute and Duke University School of Medicine, Durham, NC, USA; as Monash Health, Clayton, VIC, Australia; at Monash University, Clayton, VIC, Australia; au University of Exeter Medical School, St Luke's Campus, Exeter, UK; av West of Scotland Genetic Service, Queen Elizabeth University Hospital, Glasgow, UK; aw East Anglian Medical Genetics Service, Cambridge University Hospitals NHS Trust, Cambridge, UK; ax New Cross Hospital, Wolverhampton, UK; ay Division of Psychosocial Research and Epidemiology, The Netherlands Cancer Institute, Amsterdam, Netherlands; az Department of Urology, Erasmus Cancer Institute, Erasmus University Medical Centre, Rotterdam, Netherlands; ba Spanish National Cancer Research Center, Madrid, Spain; bb Division of Radiotherapy and Imaging, The Institute of Cancer Research, Sutton, Surrey, UK; bc Instituto Nacional de Cancer Jose de Alencar Gomes da Silva INCA, Rio de Janeiro, Brazil; bd Faculty of Medicine, University of Southampton, Southampton, UK; be Faculty of Medicine, School of Health Sciences, University of Iceland, Reykjavik, Iceland; bf Imperial College Healthcare NHS Trust, London, UK; bg HCA Pathology Laboratories, London, UK; bh University Hospital of Umeå, Umeå, Sweden; bi Churchill Hospital, Headington, Oxford, UK; bj Nuffield Department of Surgical Sciences, University of Oxford, Oxford, UK; bk Landspitali - the National University Hospital of Iceland, Reykjavik, Iceland; bl Department of Translational Medicine, Lund University, Malmö, Sweden; bm Department of Laboratory Medicine, Department of Surgery, and Department of Medicine, Memorial Sloan-Kettering Cancer Center, New York, NY, USA; bn Cancer Biology and Stem Cells Division, The Walter and Eliza Hall Institute of Medical Research, Parkville, VIC, Australia; bo International Hereditary Cancer Center, Department of Genetics and Pathology, Pomeranian Medical University in Szczecin, Szczecin, Poland; bp Department of Urology, Akershus University Hospital, Lørenskog, Norway; bq Royal Surrey County Hospital, Guildford, UK; br University College London Hospitals NHS Foundation Trust, London, UK; bs CHS National Cancer Control Center, Carmel Medical Center, Haifa, Israel; bt School of Cancer and Pharmaceutical Sciences, Faculty of Life Sciences and Medicine, King's College London, Guy's Cancer Centre, Guy's Hospital, London, UK

## Abstract

**Background:**

Lynch syndrome is a rare familial cancer syndrome caused by pathogenic variants in the mismatch repair genes *MLH1, MSH2, MSH6*, or *PMS2*, that cause predisposition to various cancers, predominantly colorectal and endometrial cancer. Data are emerging that pathogenic variants in mismatch repair genes increase the risk of early-onset aggressive prostate cancer. The IMPACT study is prospectively assessing prostate-specific antigen (PSA) screening in men with germline mismatch repair pathogenic variants. Here, we report the usefulness of PSA screening, prostate cancer incidence, and tumour characteristics after the first screening round in men with and without these germline pathogenic variants.

**Methods:**

The IMPACT study is an international, prospective study. Men aged 40–69 years without a previous prostate cancer diagnosis and with a known germline pathogenic variant in the *MLH1, MSH2*, or *MSH6* gene, and age-matched male controls who tested negative for a familial pathogenic variant in these genes were recruited from 34 genetic and urology clinics in eight countries, and underwent a baseline PSA screening. Men who had a PSA level higher than 3·0 ng/mL were offered a transrectal, ultrasound-guided, prostate biopsy and a histopathological analysis was done. All participants are undergoing a minimum of 5 years' annual screening. The primary endpoint was to determine the incidence, stage, and pathology of screening-detected prostate cancer in carriers of pathogenic variants compared with non-carrier controls. We used Fisher's exact test to compare the number of cases, cancer incidence, and positive predictive values of the PSA cutoff and biopsy between carriers and non-carriers and the differences between disease types (ie, cancer *vs* no cancer, clinically significant cancer *vs* no cancer). We assessed screening outcomes and tumour characteristics by pathogenic variant status. Here we present results from the first round of PSA screening in the IMPACT study. This study is registered with ClinicalTrials.gov, NCT00261456, and is now closed to accrual.

**Findings:**

Between Sept 28, 2012, and March 1, 2020, 828 men were recruited (644 carriers of mismatch repair pathogenic variants [204 carriers of *MLH1*, 305 carriers of *MSH2*, and 135 carriers of *MSH6*] and 184 non-carrier controls [65 non-carriers of *MLH1*, 76 non-carriers of *MSH2*, and 43 non-carriers of *MSH6*]), and in order to boost the sample size for the non-carrier control groups, we randomly selected 134 non-carriers from the *BRCA1* and *BRCA2* cohort of the IMPACT study, who were included in all three non-carrier cohorts. Men were predominantly of European ancestry (899 [93%] of 953 with available data), with a mean age of 52·8 years (SD 8·3). Within the first screening round, 56 (6%) men had a PSA concentration of more than 3·0 ng/mL and 35 (4%) biopsies were done. The overall incidence of prostate cancer was 1·9% (18 of 962; 95% CI 1·1–2·9). The incidence among *MSH2* carriers was 4·3% (13 of 305; 95% CI 2·3–7·2), *MSH2* non-carrier controls was 0·5% (one of 210; 0·0–2·6), *MSH6* carriers was 3·0% (four of 135; 0·8–7·4), and none were detected among the *MLH1* carriers, *MLH1* non-carrier controls, and *MSH6* non-carrier controls. Prostate cancer incidence, using a PSA threshold of higher than 3·0 ng/mL, was higher in *MSH2* carriers than in *MSH2* non-carrier controls (4·3% *vs* 0·5%; p=0·011) and *MSH6* carriers than *MSH6* non-carrier controls (3·0% *vs* 0%; p=0·034). The overall positive predictive value of biopsy using a PSA threshold of 3·0 ng/mL was 51·4% (95% CI 34·0–68·6), and the overall positive predictive value of a PSA threshold of 3·0 ng/mL was 32·1% (20·3–46·0).

**Interpretation:**

After the first screening round, carriers of *MSH2* and *MSH6* pathogenic variants had a higher incidence of prostate cancer compared with age-matched non-carrier controls. These findings support the use of targeted PSA screening in these men to identify those with clinically significant prostate cancer. Further annual screening rounds will need to confirm these findings.

**Funding:**

Cancer Research UK, The Ronald and Rita McAulay Foundation, the National Institute for Health Research support to Biomedical Research Centres (The Institute of Cancer Research and Royal Marsden NHS Foundation Trust; Oxford; Manchester and the Cambridge Clinical Research Centre), Mr and Mrs Jack Baker, the Cancer Council of Tasmania, Cancer Australia, Prostate Cancer Foundation of Australia, Cancer Council of Victoria, Cancer Council of South Australia, the Victorian Cancer Agency, Cancer Australia, Prostate Cancer Foundation of Australia, Asociación Española Contra el Cáncer (AECC), the Instituto de Salud Carlos III, Fondo Europeo de Desarrollo Regional (FEDER), the Institut Català de la Salut, Autonomous Government of Catalonia, Fundação para a Ciência e a Tecnologia, National Institutes of Health National Cancer Institute, Swedish Cancer Society, General Hospital in Malmö Foundation for Combating Cancer.

## Introduction

Prostate cancer is one of the major causes of morbidity and mortality in men worldwide. The importance of germline genetic variation for identifying men at increased risk of prostate cancer to enable targeted screening and early detection has become increasingly recognised.^1^

Mounting evidence suggests a moderately increased risk of prostate cancer for men with Lynch syndrome. Lynch syndrome is an autosomal, dominantly inherited, multicancer syndrome caused by a germline pathogenic variant in one of the mismatch repair genes: *MLH1, MSH2, MSH6,* or *PMS2.* The population frequency of pathogenic variants in these genes is between one (0·36%) per 279 people and one (0·035%) per 2841 people.^2^ Each gene has a different cancer incidence spectrum, with colorectal and endometrial cancers being the predominant phenotype. These pathogenic variants are also associated with an increased risk of other cancers including those of the ovary, stomach, small bowel, ureter, kidney, and brain.^3^, ^4^

Lynch syndrome has been reported to increase risk of prostate cancer by two-to-ten times.^5^, ^6^, ^7^, ^8^, ^9^ Most evidence has come from studies of men with prostate cancer from families with mismatch repair pathogenic variants. Tumour testing has shown loss of expression of mismatch repair proteins and microsatellite instability.^7^, ^10^ However, mismatch repair deficiency does not conclusively prove that a tumour is caused by a germline variant. Other studies have attempted to estimate risk of prostate cancer in patients with Lynch syndrome by looking at the incidence of prostate cancer within families with Lynch syndrome.^4^, ^5^, ^7^, ^11^, ^12^, ^13^, ^14^ These studies are restricted by their size and design, often including men whose pathogenic variant status is unconfirmed, but they generally support an increased risk of prostate cancer.^9^ Other studies have not found an increased risk of prostate cancer in association with Lynch syndrome.^10^, ^15^ A meta-analysis reported a 2·13 times increased risk of prostate cancer and supported prostate cancer being considered part of Lynch syndrome.^9^ An association with higher grade tumours and younger age of onset has been reported in some studies,^6^, ^7^, ^16^, ^17^ while others have found no such associations.^8^ Most studies have been underpowered to observe differences by a specific mismatch repair gene, but there is more evidence of an increased risk of prostate cancer associated with pathogenic variants in *MSH2* than the other genes.^3^, ^5^, ^7^, ^8^, ^13^, ^14^

**Research in context**

**Evidence before this study**

We did not do a formal systematic review when planning the design of this trial because there were no published studies assessing prostate cancer screening in men with mismatch repair pathogenic variants. Evidence was assimilated about the risk of prostate cancer associated with the mismatch repair genes. These studies took two approaches: those that assessed the tumours of men with prostate cancer from families with known pathogenic variants, and those that assessed incidence in families with known germline variants. However, not all men in these studies had their germline mutation status confirmed, limiting their design. A published meta-analysis reported a two times increased risk of prostate cancer for men with known mutations, associated with higher-grade tumours and younger age of onset.

**Added value of this study**

In this international prospective screening study of 828 men from families with confirmed pathogenic variants in mismatch repair genes, of whom 186 had a family history of prostate cancer, we found that after one screening round a higher incidence of prostate cancer was detected in men with *MSH2* and *MHS6* pathogenic variants compared with age-matched non-carrier controls. Additionally, we identified that *MSH2* carriers were diagnosed at a non-significantly younger age and had more clinically significant disease at diagnosis compared with non-carriers. Therefore, these data add evidence that prostate screening in this higher-risk context has potential to detect tumours that are highly likely to need treatment based on national and international guidelines without the limitations of over-detection seen in general population screening programmes.

**Implications of all the available evidence**

Our findings support the use of targeted prostate-specific antigen screening in men with mismatch repair gene pathogenic variants to successfully detect clinically significant prostate cancers. To our knowledge, the IMPACT consortium has the largest cohort of men with mismatch repair pathogenic variants being screened and followed up, and subsequent screening rounds and detection of incident cancers will be important to confirm the optimal screening interval for early detection of clinically important tumours and prevention of metastatic events. As the use of immunotherapies within the management of prostate cancer increases, the IMPACT study has provided valuable evidence about the risk of prostate cancer, tumour characteristics, and long-term clinical outcomes in men with mismatch repair gene pathogenic variants who go on to develop prostate cancer. Testing for mismatch repair variants will likely become routine practice at diagnosis over the coming years.

The prostate-specific antigen (PSA) test is the most effective prostate cancer biomarker; however, its limitations have been well documented. Data from long-term follow-up in the European Randomised Study of Screening for Prostate Cancer (ERSPC)^18^, ^19^ and Prostate, Lung, Colorectal and Ovary screening study (PLCO)^20^, ^21^, ^22^ are similar, indicating a 25–32% decrease in death from prostate cancer when PSA testing is used in a screening context.^23^ However, authoritative groups do not support routine PSA screening for the general population because of harms due to overdetection and treatment. Most screening advisory bodies, including the American Cancer Society and European Association of Urology (EAU), recommend PSA screening for men with a strong family history of prostate cancer (ie, a first degree relative who is diagnosed below the age of 70 years, or multiple relatives being diagnosed on the same side of the family). The EAU updated their guidelines to include annual PSA screening in men with *BRCA2* pathogenic variants from age 40 years, based on the results of the *BRCA1* and *BRCA2* cohorts of the IMPACT study (Identification of Men with a genetic predisposition to ProstAte Cancer: Targeted screening in men at higher genetic risk and controls).^24^, ^25^, ^26^

No international consensus exists on screening for prostate cancer in men with Lynch syndrome. The updated 2019 National Comprehensive Cancer Network (NCCN) guidelines^27^ recommended consideration of tumour testing for homologous recombination mutations and microsatellite instability or deficient mismatch repair in men with regional or metastatic prostate cancer. Germline testing should be offered to all newly diagnosed men with NCCN high-risk, very high-risk, regional, or metastatic prostate cancer. The higher risk and possible predisposition to aggressive disease is similar to the risk of prostate cancer associated with pathogenic variants in *BRCA2.*^9^, ^25^ A confirmed risk of prostate cancer in men with mismatch repair pathogenic variants would offer the opportunity for targeted screening to enable earlier detection. To date, no studies have been published assessing targeted screening for prostate cancer in men with known pathogenic variants in the mismatch repair genes.

The IMPACT study was established in 2005 to assess targeted PSA screening in men with *BRCA1* or *BRCA2* pathogenic variants.^24^, ^25^ Using the established IMPACT infrastructure, the protocol was extended in 2012 to include men from families with *MLH1, MSH2,* and *MSH6* pathogenic variants. *PMS2* was not included due to the paucity of data supporting an increased risk of prostate cancer. Here, we report the results of the first screening round for all men enrolled into the mismatch repair cohort of IMPACT. We aimed to assess the usefulness of PSA screening and determine the incidence of prostate cancer, positive predictive value (PPV) of biopsy, biopsy rates, and tumour characteristics. Our hypothesis was that men with pathogenic variants in the mismatch repair genes would have a significantly increased risk of prostate cancer compared with non-carrier controls.

## Methods

### Study design and participants

The IMPACT study is an international prospective, targeted, prostate cancer screening study in men at a genetically higher risk of prostate cancer than age-matched controls.^28^ The original protocol was designed to screen men with *BRCA1* and *BRCA2* pathogenic variants, and amended on June 29, 2012, to include a mismatch repair cohort to undergo the same study algorithm.

For the mismatch repair cohort, we recruited men from genetics and urology clinics from 34 centres in eight countries (Australia, Israel, Italy, Norway, Portugal, Spain, the UK, and the USA; appendix pp 4–5). Men aged 40–69 years were eligible for the study if they had undergone genetic testing and tested positive or negative for a known familial pathogenic variant (*MSH1, MSH2*, or *MSH6*), or if they were at 50% risk of inheriting a pathogenic variant (ie, a first degree relative has tested positive for a known variant) but had not yet undergone testing. Men who were at risk and who had yet to be tested were tested as part of the study and allocated to the appropriate analysis group; this result was not disclosed to the participants, and is not planned to be disclosed unless the participant requests it. Men were excluded if they were known to have prostate cancer or if they had a previous cancer diagnosis with a prognosis of less than 5 years survival.

The study was approved by the UK West-Midlands Research and Ethics Committee (reference 05/MRE07/25), and subsequently by each participating institution's local committee. All participants provided written informed consent and interim analyses are presented to the Independent Data and Safety Monitoring Committee twice a year. The study protocol is available online.

### Procedures

Men with a pathogenic variant were age-matched with non-carrier controls in a 1:1 ratio. Men were age-matched to within 5 years of age of their allocated carrier.

Participants underwent a PSA blood test at enrolment and PSA was measured at their local clinical laboratory to determine clinical action. For participants with a PSA concentration of higher than 3·0 ng/mL, transrectal, ultrasound-guided, prostate biopsy was recommended. Decision to biopsy was based on this single PSA level, and the screening was not repeated unless clinically indicated. A concurrent serum sample was taken for PSA quality assurance testing and was shipped to HL's laboratory (Wallenberg Research Laboratory, SUS Skånes University Hospital, Malmo, Sweden) for analysis using the ProStatus PSA Free/Total DELFIA assay (PerkinElmer Life and Analytical Sciences, Boston, MA, USA). The laboratory technicians who processed the samples were masked to participant clinical outcome data and genetic status; these data will be subject to future analyses.

**Figure 1:**
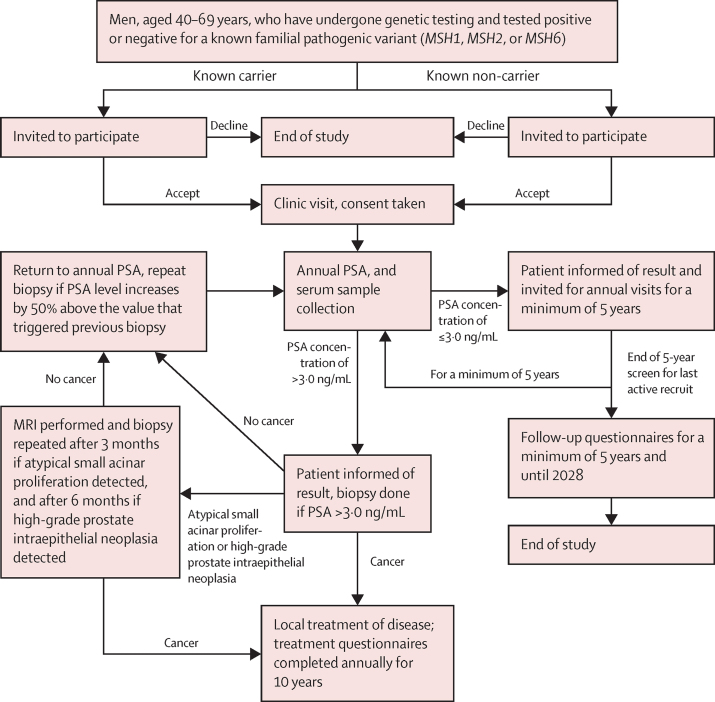
Study algorithm

Centres were requested to follow a standard 12 core biopsy protocol. The IMPACT protocol was written before the routine use of MRI in the diagnostic pathway, but MRI data were collected where available. Participants with a benign prostate biopsy (classified as no cancer) continued annual PSA screenings and follow-up (figure 1). The local histopathologist at each centre reported the biopsy outcome to guide treatment in accordance with local guidelines. Cancers were deemed to be clinically significant if classified as intermediate-risk (PSA concentration of 10–20 ng/mL, Gleason score of 7, or TNM classification of T2b) or high-risk (PSA concentration of >20 ng/mL, Gleason score of ≥8, or TNM classification of ≥T2c) as defined using the National Institute for Health and Care Excellence (NICE) guidelines. Whenever high-grade prostate intraepithelial neoplasia or atypical small acinar proliferation was detected, the biopsy was repeated after 3 months if atypical small acinar proliferation was detected and after 6 months if high-grade prostate intraepithelial neoplasia was detected. Participants with a PSA concentration of 3·0 ng/mL or less will undergo annual PSA screening for a minimum of 5 years. Participants with a PSA concentration higher than 3·0 ng/mL and a negative biopsy will continue annual PSA testing, with the biopsy to be repeated if their PSA concentration increases by more than 50%. All participants will be followed up for at least 10 years to assess cancer incidence and prostate cancer-specific mortality and morbidity. A central pathological review is ongoing as part of our quality control measures and will be reported after the full 5 years of screening have been completed.

The *MSH6* carrier cohort did not reach its recruitment target and so analyses were done with the numbers available. Because the control cohorts did not reach recruitment target, samples from men in the *BRCA1* and *BRCA2* non-carrier control cohort of IMPACT were randomly selected by members of the laboratory team from a plate of anonymised extracted DNA samples to supplement the control group. The selected mismatch repair genes were sequenced from germline DNA using targeted next-generation sequencing and analysed for pathogenic variants using Agilent SureCall (version 4.2.1; Santa Clara, CA, USA).

### Outcomes

The primary endpoint was to determine the incidence, stage, and pathology of screen-detected prostate cancer in carriers of mismatch repair pathogenic variants compared with non-carrier controls. Secondary endpoints were to determine age-specific PSA concentrations in carriers of pathogenic variants in mismatch repair genes versus age-matched non-carrier controls and men in two population-based screening studies (ERSPC and PLCO);^18^, ^19^, ^20^ to determine a profile of PSA concentration and its predictive value for the development of prostate cancer in carriers of mismatch repair pathogenic variants using 5 years, or more, of annual follow-up compared with the control populations (ie, age-matched non-carrier and population-based studies); to assess the sensitivity and specificity of new serum and urine markers of prostate cancer in carriers of mismatch repair pathogenic variants; to develop microarrays to determine the genetic profile of prostate cancers occurring in carriers of mismatch repair pathogenic variants; and to characterise the genomic and biological profiles in samples from carriers with mismatch repair pathogenic variants and changes related to prostate cancer in those individuals. All secondary endpoints require the full 5 years of PSA screening to be completed and will be reported as part of future analyses.

### Statistical analysis

We hypothesised that men with pathogenic variants in the mismatch repair genes would have at least a two times increased risk of prostate cancer compared with non-carrier controls. IMPACT has been powered to detect a two-times relative risk of prostate cancer over 5 years of screening, with 80% power at an α level of less than 0·01. The target sample was 190 men aged 40–69 years from each of the following six groups: *MLH1, MSH2*, and *MSH6* germline pathogenic variant carriers and non-carriers. We ensured that the same proportion of carriers and non-carrier controls were within each age group of 40–49, 50–59, and 60–69 years. We also ensured that mean and median ages were within 5 years for each cohort versus their control group. The number of missing PSA readings was low (n=4) and in post-hoc analyses these were counted as negative—ie, did not trigger a biopsy.

We used Fisher's exact test to compare the number of cases and incidence of prostate cancer, and PPV of the PSA cutoff and biopsy for prostate cancer between carriers and non-carriers and the differences between disease types (ie, cancer *vs* no cancer and clinically significant cancer *vs* no cancer). We assessed screening outcomes and tumour characteristics by pathogenic variant status. We used Student's *t* test to compare mean ages and PSA readings. We used the Mann-Whitney *U* test to compare median ages and PSA readings. All statistical tests were-two tailed and p values of less than 0·05 were considered to be significant. For assumption checking, we used 95% CIs for proportions and χ^2^ tests, for both we used exact CIs where appropriate. We did a sensitivity analysis to assess the robustness of the results to changes in biopsy compliance rates in the non-carrier controls. We assumed that the biopsy rates in the non-carrier controls was the same as among the carrier group, and recalculated the number of cancers that could have been present in the non-carriers assuming that any additional biopsies resulted in a cancer outcome and compared cancer incidence.

Interim analyses are planned for when all participants have completed 3 years of screening. The final analysis will be completed after all participants have completed a minimum of 5 years of screening.

We did all statistical analysis using Graphpad (version 9.0.2) and Stata (version 16.1). This study is registered with ClinicalTrials.gov, NCT00261456.

### Role of the funding source

The study sponsor, The Institute of Cancer Research, has oversight of study design and conduct and had no role in the data collection, data analysis, data interpretation, or writing of the report. The funders of the study had no role in study design, data collection, data analysis, data interpretation, or writing of this report.

## Results

**Figure 2:**
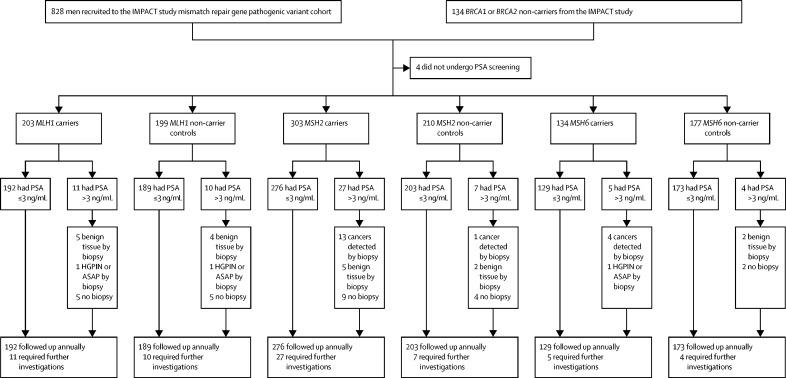
Trial profile *BRCA1* and *BRCA2* non-carrier controls were included in every non-carrier control group, but only counted once in the total cohort number, therefore the sum of each genetic cohort does not equal the total. ASAP=atypical small acinar proliferation. HGPIN=high-grade prostate intraepithelial neoplasia.

Between Sept 28, 2012, and March 1, 2020, 962 men were recruited, of whom 828 (86%) were recruited as part of the mismatch repair pathogenic variant cohort (644 [78%] carriers of a mismatch repair pathogenic variant [204 (32%) carriers of *MLH1,* 305 (47%) carriers of *MSH2,* and 135 (21%) carriers of *MSH6*] and 184 [22%] non-carrier controls [65 (35%) non-carriers of *MLH1*, 76 (41%) non-carriers of *MSH2,* and 43 (23%) non-carriers of *MSH6*]; appendix pp 4–5) and 134 (14%) were randomly selected from the *BRCA1* and *BRCA2* non-carrier cohort of the IMPACT study and screened for pathogenic variants in the mismatch repair genes and their data were used to supplement the control groups (figure 2). Men were predominantly of European ancestry (899 [94%] of 953 with available data) and most had a technical or vocational qualification (180 [20%] of 910 with available data) or had graduated university (372 [41%] of 910; table 1). Median age at enrolment was 53 years (IQR 46–59). 208 (22%) participants reported previous urinary symptoms, 318 (38%) had previously had a PSA test (no significant difference between carriers and non-carrier controls), and 186 (19%) had at least one first-degree or second-degree relative with prostate cancer (self-reported), with significantly more non-carrier controls reporting a family history of prostate cancer in the *MSH2* (p=0·028) and *MLH1* (p=0·0044) groups than carriers; for the *MSH6* group, there was no significant difference between carriers and non-carrier controls (p=0·081; table 1).

**Table 1:** Baseline demographic and clinical characteristics

	Total cohort (n=962*)	***MLH1* carriers (n=204)**	***MLH1* non-carrier controls (n=199)**	***MSH2* carriers (n=305)**	***MSH2* non-carrier controls (n=210)**	***MSH6* carriers (n=135)**	***MSH6* non-carrier controls (n=177)**
**Age, years**							
40–49	369 (38%)	76 (37%)	67 (34%)	133 (44%)	77 (37%)	50 (37%)	62 (35%)
50–59	358 (37%)	78 (38%)	79 (40%)	101 (33%)	76 (36%)	54 (40%)	70 (40%)
60–69	235 (24%)	50 (25%)	53 (27%)	71 (23%)	57 (27%)	31 (23%)	45 (25%)
Median	53 (46–59)	52 (46–59)	54 (46–54)	51 (45–59)	54 (46–60)	54 (46–60)	53 (46–59)
Mean	52·8 (8·3)	52·7 (8·2)	53·9 (8·2)	51·9 (8·2)	53·5 (8·6)	53·6 (8·2)	52·9 (8·0)
p value for difference in mean between carriers and non-carrier controls	NA	0·15	..	0·036	..	0·42	..
**Qualifications**							
No qualifications	61/910 (6%)	15/196 (8%)	16/187 (9%)	11/286 (4%)	18/198 (9%)	8/129 (6%)	13/168 (8%)
Attended school up to age 16 years	172/910 (19%)	34/196 (17%)	34/187 (18%)	60/286 (21%)	38/198 (19%)	20/129 (16%)	28/168 (17%)
Attended school up to age 18 years or College degree	82/910 (9%)	18/196 (9%)	24/187 (13%)	20/286 (7%)	26/198 (13%)	8/129 (6%)	24/168 (14%)
Technical or vocational qualification	180/910 (20%)	32/196 (16%)	47/187 (25%)	53/286 (19%)	45/198 (23%)	23/129 (18%)	40/168 (24%)
University graduate	372/910 (41%)	81/196 (41%)	63/187 (34%)	127/286 (44%)	71/198 (36%)	64/129 (50%)	60/168 (36%)
Other qualification	43/910 (5%)	16/196 (8%)	3/187 (2%)	15/286 (5%)	0	6/129 (5%)	3/168 (2%)
Unknown	52	8	12	19	12	6	9
**Ethnicity**							
European ancestry	899/953 (94%)	176/201 (88%)	192/199 (96%)	287/304 (94%)	206/209 (99%)	130/132 (98%)	174/176 (99%)
Black African or Black Caribbean ancestry	5/953 (1%)	1/201 (<1%)	0	4/304 (1%)	0	0	0
Asian ancestry	37/953 (4%)	18/201 (9%)	6/199 (3%)	10/304 (3%)	2/209 (1%)	1/132 (1%)	2/176 (1%)
Mixed	12/953 (1%)	6/201 (3%)	1/199 (1%)	3/304 (1%)	1/209 (<1%)	1/132 (1%)	0
Unknown	9	3	0	1	1	3	1
**Family history of prostate cancer (self-reported)**†							
Yes	186 (19%)	31 (15%)	49 (25%)	56 (18%)	58 (28%)	29 (21%)	47 (27%)
No	776 (81%)	173 (85%)	150 (75%)	249 (82%)	152 (72%)	106 (79%)	130 (73%)
p value for difference between carriers and non-carrier controls	NA	0·0044	..	0·028	..	0·081	..
**Previous urinary symptoms**					**..**		
Yes	208/949 (22%)	43/203 (21%)	53 (27%)	55/302 (18%)	42/207 (20%)	33/131 (25%)	42/175 (24%)
No	741/949 (78%)	160/203 (79%)	146 (73%)	247/302 (82%)	165/207 (80%)	98/131 (75%)	133/175 (76%)
Unknown	13	1	0	3	3	4	2
p value for difference between carriers and non-carrier controls	NA	0·24	..	0·57	..	0·89	..
**Previous PSA test**							
Yes	318/834 (38%)	71/168 (42%)	76/181 (42%)	92/266 (35%)	72/189 (38%)	45/116 (39%)	68/154 (44%)
No	516/834 (62%)	97/168 (58%)	105/181 (58%)	174/266 (65%)	117/189 (62%)	71/116 (61%)	86/154 (56%)
Unknown	128	36	18	39	21	19	23
p value for difference between carriers and non-carrier controls	NA	0·99	..	0·49	..	0·39	..

Data are n (%), n/N (%), n, median (IQR), mean (SD), or p value. PSA=prostate-specific antigen.

* *BRCA1* and *BRCA2* non-carrier controls were included in every non-carrier control group but only counted once in the total cohort number; therefore, the sum of each genetic cohort does not equal the total.

† First-degree or second-degree relative with prostate cancer.

The overall study population comprised 962 participants, including the 134 non-carriers who were counted within each of the non-carrier control groups. The *MSH6* cohort did not reach target capacity because of the rarity of indentified carriers.

Among 962 men, 958 (>99%) baseline PSA screening results were available, four (<1%) PSA screening results were missing or not obtained and these were counted as negative (ie, did not trigger a biopsy); therefore, this did not affect the overall result (one *MLH1* carrier, two *MSH2* carriers, and one *MSH6* carrier). 56 (6%) of 962 men had a PSA reading higher than 3·0 ng/mL (median 5·1 ng/mL [IQR 3·8–11·1]), requiring referral for a prostate biopsy, of whom 35 (63%) had a biopsy (figure 2, table 2). 21 (37%) of 56 men declined prostate biopsy due to concurrent health conditions (n=2), urologist repeating PSA reading before prostate biopsy resulting in a reading of 3·0 ng/mL or lower (n=9), undergoing an MRI with no abnormalities (n=4), or men changing their mind (n=6).

**Table 2:** Participants summarised by mutation status, PSA summary data, and PPV of PSA and biopsy

			Total cohort (n=962*)	MLH1 **carriers (n=204)**	MLH1 **non-carrier controls (n=199)**	MSH2 **carriers (n=305)**	MSH2 **non-carrier controls (n=210)**	MSH6 **carriers (n=135)**	MSH6 **non-carrier controls (n=177)**
Total PSA screenings done at baseline			958	203	199	303	210	134	177
Median PSA concentrations, ng/mL			0·8 (0·6 to 1·4)	0·9 (0·6 to 1·4)	0·8 (0·6 to 1·4)	0·8 (0·6 to 1·4)	0·9 (0·6 to 1·5)	0·8 (0·6 to 1·4)	0·8 (0·5 to 1·4)
PSA concentration >3·0 ng/mL			56 (6%)	11 (5%)	10 (5%)	27 (9%)	7 (3%)	5 (4%)	4 (2%)
Biopsies†			35 (4%)	6 (3%)	5 (3%)	18 (6%)	3 (1%)	5 (4%)	2 (1%)
	Benign tumour		14	5	4	5	2	0	2
	ASAP or HGPIN		3	1	1	0	0	1	0
	Malignant tumour—ie, prostate cancer incidence with PSA >3·0 ng/mL		18 (1·9% [1·1 to 2·9])	0	0	13 (4·3% [2·3 to 7·2])	1 (0·5% [0·0 to 2·6])	4 (3·0% [0·8 to 7·4])	0
		Difference between carriers and non-carrier controls	NA	0	..	3·8% (1·3 to 6·2)	..	3·0% (0·1 to 5·8)	..
		p value	NA		..	0·011	..	0·034	..
	Clinically significant prostate cancer incidence with PSA >3·0 ng/mL		14 (1·5% [0·8 to 2·4])	0	0	11 (3·6% [1·8 to 6·4])	0	3 (2·2%[0·5 to 6·4])	0
		Difference between carriers and non-carrier controls	NA	NA	..	3·6% (1·5 to 5·7)	..	2·2% [−0·3 to 4·7)	..
		p value	NA	NA	..	0·0037	..	0·080	..
PPV of biopsy‡			51·4% (34·0 to 68·6)	0	0	72·2% (46·5 to 90·3)	33·3% (0·8 to 90·6)	80·0% (28·4 to 99·5)	0
	Difference between carriers and non-carrier controls		NA	0	..	38·9% (18·3 to 96·1)	..	80·0% (44·9 to 115·1)	..
	p value		NA	NA	..	0·25	..	0·14	..
PPV of PSA >3·0 ng/mL requiring action§			32·1% (20·3 to 46·0)	0	0	48·1% (28·7 to 68·1)	14·3% (0·4 to 57·9)	80·0% (28·4 to 99·5)	0
	Difference between carriers and non-carrier controls		NA	0	..	33·9% (1·8 to 65·9)	..	80·0% (44·9 to 115·1)	..
	p value		NA	NA	..	0·20	..	0·048	..

Data are n (%), median (IQR), n with incidence and 95% CI in parentheses, or incidence with 95% CI in parentheses. ASAP=atypical small acinar proliferation. HGPIN=high-grade prostate intraepithelial neoplasia. NA=not applicable. PPV=positive predictive value.

* *BRCA1* and *BRCA2* non-carrier controls were included in every non-carrier control group, but only counted once in the total cohort number; therefore, the sum of each genetic cohort does not equal the total.

† Not including off-protocol biopsies (in participants with PSA <3·0 ng/mL).

‡ PPV of biopsy is number of cancers diagnosed divided by the number of biopsies performed.

§ PPV of PSA >3ng/mL requiring action is number of cancers diagnosed divided by number of PSA readings of >3·0 ng/mL.

Of the 35 biopsies performed, 18 (51%) indicated the presence of cancer and were in 13 (4%) of 305 *MSH2* carriers, four (3%) of 135 *MSH6* carriers, and one (<1%) of 210 *MSH2* non-carrier controls. No cancers were diagnosed in the *MLH1* carriers, *MLH1* non-carrier controls, or *MSH6* non-carrier controls. The number of biopsy cores taken across all biopsies ranged from six to 33, age at biopsy ranged from 40 to 69 years, and no significant differences in these biopsy characteristics were seen between groups (table 3). Higher compliance with biopsy was observed in *MSH2* carriers than in non-carrier controls (18 [67%] of 27 *vs* three [43%] of seven; p=0·39) and in *MSH6* carriers than in non-carrier controls (five [100%] of five *vs* two [50%] of four; p=0·17; table 3), although these differences were not significant.

**Table 3:** Summary of characteristics of men who underwent biopsies after their baseline PSA screen

	Total cohort*	MLH1 **carriers**	MLH1 **non-carrier controls**	**p value**	MSH2 **carriers**	MSH2 **non-carrier controls**	**p value**	MSH6 **carriers**	MSH6 **non-carrier controls**	**p value**
Total biopsies	35	6	5		18	3		5	2	
Biopsy compliance	35/56 (63%)	6/11 (55%)	5/10 (50%)	>0·99	18/27 (67%)	3/7 (43%)	0·39	5/5 (100%)	2/4 (50%)	0·17
PSA concentration that triggered biopsy, ng/mL	5·1 (3·80–11·1)	3·9 (3·5–5·4)	4·2 (3·6–9·4)	0·72	5·8 (3·8–20·6)	5·1 (3·4–5·3)	0·45	7·8 (4·0–9·9)	4·3 (3·4–5·1)	0·43
Age at biopsy, years	61 (56–64)	60 (55–64)	62 (62–64)	0·35	60 (53–64)	64 (62–66)	0·13	64 (59–67)	63 (62–64)	0·86
Time between PSA screening and biopsy, days	91 (54–148)	92 (39–169)	87 (64–96)	0·70	105 (43–179)	87 (80–256)	0·66	89 (77–120)	84 (80–87)	0·57
Biopsy cores taken	12 (12–14)	12 (10–23)	12 (10–14)	>0·99	13 (12–15)	10 (8–12)	0·07	13 (11–13)	10 (8–12)	0·27

Data are n, n/N (%), or median (IQR).

* *BRCA1* and *BRCA2* non-carrier controls were included in every non-carrier control group but only counted once in the total cohort number; therefore, the sum of each genetic cohort does not equal the total.

Three participants had off-protocol biopsies (ie, with PSA concentrations of <3·0 ng/mL) after their baseline PSA screening. Two malignant biopsies were identified in these participants: an *MSH2* carrier with an abnormal rectal examination (PSA concentration of 0·85 ng/mL, Gleason score of 5+4, and TNM classification T-stage of T3a) and an *MLH1* non-carrier control (PSA concentration of 2·97 ng/mL, Gleason score of 3+3, and TNM classification T-stage of T2c; table 4). The third participant, an *MSH2* carrier with a PSA concentration of 2·98 ng/mL), had benign tissue on biopsy. Additionally, three men (one *MLH1* carrier, one *MLH1* non-carrier control, and one *MSH6* carrier) had either atypical small acinar proliferation or high-grade prostate intraepithelial neoplasia.

**Table 4:** Clinical features of all 18 on-protocol and two off-protocol participants for whom prostate cancer was diagnosed in the first screening round

	**Age at diagnosis, years**	**Diagnostic PSA concentration, ng/mL**	**Previous PSA test**	**Family history of prostate cancer**	**Previous urinary symptoms**	**Risk scores**					**Biopsy**		**Treatment**
						Cancer risk*	Gleason score†	T stage†	N stage†	M stage†	Total cores taken	Total cancer cores	
**On-protocol cancers (PSA >3 ng/mL)**													
*MSH2* non-carrier	66	5·3	No	No	No	Low	3+3	T2a	Nx	M0	10	1	Radical prostatectomy
*MSH2* carrier	58	3·05	Yes	No	No	Low	3+3	T1c	N0	M0	12	5	Active surveillance
*MSH2* carrier	57	11·05	Yes	Yes	No	Intermediate	NA	NA	NA	NA	NA	NA	Radical prostatectomy plus hormone therapy
*MSH2* carrier	69	10·0	Yes	No	Yes	Intermediate	4+3	T2a	NA	NA	14	3	Non-surgical treatment
*MSH2* carrier	60	31·1	No	No	No	High	4+3	T3	NA	NA	NA	NA	Radiotherapy
*MSH2* carrier	64	89·7	No	No	No	High	4+5	T3b	N1	M1b	NA	NA	Non-surgical treatment
*MSH2* carrier	64	20·6	No	Yes	No	High	4+5	T3b	NA	NA	12	5	Non-surgical treatment
*MSH2* carrier	40	22·5	No	No	No	High	4+3	T2a	Nx	M0	NA	NA	Radical prostatectomy
*MSH2* carrier	58	5·8	No	No	No	Low	3+3	NA	NA	NA	20	5	Active surveillance
*MSH2* carrier	44	3·3	No	No	No	High	3+3	T2c	N0	M0	28	4	Active surveillance
*MSH2* carrier	61	14·0	No	No	No	Intermediate	3+4	T2a	N0	M0	15	2	Non-surgical treatment
*MSH2* carrier	66	13·0	Yes	No	No	High	3+4	T3b	Nx	Mx	14	5	Radical prostatectomy
*MSH2* carrier	48	3·5	Unknown	No	No	High	4+3	T2c	N0	Mx	18	3	Radical prostatectomy
*MSH2* carrier	65	29·0	No	No	Yes	High	4+4	T3a	N0	Mx	NA	NA	Radical prostatectomy plus hormone therapy
*MSH6* carrier	67	7·8	No	No	No	High	4+4	T2b	N0	Mx	10	1	Active surveillance
*MSH6* carrier	55	11·1	No	No	No	High	3+4	T2c	N0	M0	13	4	Radical prostatectomy
*MSH6* carrier	62	4·5	No	No	No	High	3+4	T2c	Nx	M0	NA	NA	Radical prostatectomy
*MSH6* carrier	66	3·4	Yes	No	Yes	Low	3+3	NA	NA	NA	12	1	Active surveillance
**Off-protocol cancers (PSA ≤3·0 ng/mL)**													
*MSH2* carrier	49	0·85	No	No	No	High	5+4	T3a	N0	M0	12	6	Radical prostatectomy plus hormone and radiotherapy
*MLH1* non-carrier	66	2·97	Yes	No	Yes	High	3+3	T2c	N0	M0	NA	NA	Radical prostatectomy

NA=not available.

* Using National Institute for Health and Care Excellence guidelines.

† Gleason score and TNM stage were taken from the participants' most recent histology data.

Overall prostate cancer incidence for the baseline screening, using a PSA threshold of more than 3·0 ng/mL, was 1·9% (18 of 962; 95% CI 1·1–2·9; table 2). The incidence among *MSH2* carriers was 4·3% (13 of 305; 95% CI 2·3–7·2) compared with 0·5% (one of 210; 0·0–2·6) in *MSH2* non-carrier controls; a difference of 3·8% (95% CI 1·3–6·2; p=0·011). The incidence among *MSH6* carriers was 3·0% (four of 135; 95% CI 0·8–7·4) compared with 0% (none of 177) among *MSH6* non-carrier controls; a difference of 3·0% (95% CI 0·1–5·8; p=0·034). When looking at the incidence of clinically significant prostate cancer, the incidence among *MSH2* carriers was 3·6% (11 of 305; 95% CI 1·8 to 6·4) compared with 0% (none of 210) among *MSH2* non-carrier controls (p=0·0037). The incidence among *MSH6* carriers was 2·2% (three of 135; 95% CI 0·5–6·4) compared with 0% (none of 177) among *MSH6* non-carrier controls (p=0·080; table 2).

In a sensitivity analysis, we found that if the biopsy compliance rate for the carrier cohorts was applied to the non-carrier control cohorts, an additional cancer might have been identified in the *MSH2* and *MSH6* non-carrier control cohorts. Under this scenario of a 67% biopsy compliance rate in the control cohort, the prostate cancer incidence would then be 1·0% (two of 210; 95% CI 0·1–3·4) in *MSH2* non-carrier controls (*vs* 4·3% [13 of 305; 2·3–7·2] in *MSH2* carriers), with a difference in incidence of 3·3% (0·7–5·9; p=0·032). In the *MSH6* non-carrier control cohort, under a scenario of a 100% biopsy compliance rate, the prostate cancer incidence would then be 0·6% (one of 177; 95% CI 0·0–3·1; *vs* 3·0% [four of 135; 0·8–7·4] in *MSH6* carriers), with a difference in incidence of 2·4% (0·7–5·5; p=0·17).

The overall PPV of biopsy using a PSA threshold of 3·0 ng/mL (ie, number of prostate cancers identified divided by number of prostate biopsies) was 51·4% (18 of 35; 95% CI 34·0–68·6; table 2). When separated by genetic status, PPV in *MSH2* carriers was 72·2% (13 of 18; 46·5–90·3), in *MSH2* non-carrier controls was 33·3% (one of three; 0·8–90·6), and in *MSH6* carriers was 80·0% (four of five; 28·4–99·5). We could not calculate PPV for the *MSH6* non-carrier controls, *MLH1* carriers, and *MLH1* non-carrier controls because no cases were detected in these groups. There were no significant differences between carriers and non-carriers of each gene (table 2).

The overall PPV of PSA concentration higher than 3·0 ng/mL (ie, number of prostate cancers identified divided by number of PSA readings of more than 3·0 ng/mL) at detecting prostate cancer was 32·1% (18 of 56; 95% CI 20·3–46·0). Similarly, when separated by genetic status, the PPV in *MSH2* carriers was 48·1% (13 of 27; 28·7–68·1), in *MSH2* non-carriers was 14·3% (one of seven; 0·4–57·9), and in *MSH6* carriers was 80·0% (four of five; 28·4–99·5). We could not calculate PPV for the *MSH6* non-carrier controls, *MLH1* carriers, and *MLH1* non-carrier controls because no cases were detected in these groups. There was a significant difference in PPV between *MSH6* carriers and non-carrier controls (p=0·048) and no significant difference between *MSH2* carriers and non-carrier controls (p=0·20).

In *MSH2* carriers, the mean age at diagnosis was 58 years (SD 9) compared with 66 years (SD 0) in the non-carriers (p=0·40). Mean PSA concentration at prostate cancer diagnosis was 19·7 ng/mL (SD 8·9) in *MSH2* carriers compared with 5·3 ng/mL (SD 0) in *MSH2* non-carrier controls (p=0·56). In *MSH6* carriers the mean age at biopsy was 63 years (SD 5) and the mean PSA concentration at diagnosis was 6·7 ng/mL (SD 3.5). Only three (17%) of 18 men who were diagnosed with on-protocol prostate cancer reported urinary symptoms before diagnosis (two *MSH2* carriers and one *MSH6* carrier) and five (28%) had previously had a PSA test before study entry (four *MSH2* carriers and one *MSH6* carrier; table 4). Using the NICE classification, intermediate-risk or high-risk tumours were observed in 11 (85%) of 13 *MSH2* carriers with a diagnosis versus none of one *MSH2* non-carrier control with a diagnosis. We found no significant difference between genetic status and NICE classification in the *MSH2* cohort (p=0·43). One *MSH2* carrier had nodal involvement and metastatic disease at diagnosis. Three (75%) of four cancers detected in *MSH6* carriers were classified as high risk, but no cancers were detected in *MSH6* non-carrier controls to enable a comparison. There was no specific pathogenic variant or gene region associated with the cancers diagnosed in the *MSH2* and *MSH6* carriers (data not shown).

## Discussion

Here, we present the results of the first screening round of the mismatch repair cohort enrolled in the IMPACT study. With mismatch repair germline pathogenic variants being relatively rare, the success of IMPACT has been in the use of an existing international consortium. We found a significantly higher incidence of prostate cancer in men with pathogenic variants in *MSH2* and *MSH6* compared with non-carrier controls, supporting that the risk of prostate cancer is increased with Lynch syndrome, and specifically with *MSH2* and *MSH6*.

Since the initial design of the IMPACT study in 2005, multiparametric MRI has increasingly become a standard part of the diagnostic pathway, and only men with targetable MRI lesions proceed to biopsy.^29^ In our study, four men with PSA concentrations of more than 3·0 ng/mL and normal MRIs were not put forward for biopsy by their local urology team, despite the protocol advising biopsy in all men with a PSA concentration of more than 3·0 ng/mL. Compliance with biopsy was 63% overall, which is slightly lower than the 81% compliance in the *BRCA1* and *BRCA2* cohort of IMPACT after the baseline screen^24^ and the 86% compliance in the ERSPC, and higher than the 31·5% compliance in the PLCO studies after baseline PSA screen.^18^, ^19^, ^20^, ^21^, ^22^ By 3 years of follow-up in the PLCO study, 64% of participants had a biopsy, and therefore a similar level of compliance might be observed in subsequent screening rounds of IMPACT in the current cohort. We found no significant differences in biopsy compliance between carriers and non-carriers in each gene group.

56 (6%) of 962 men had a positive PSA test (>3·0 ng/mL), which is lower than the 16·2% (range 11·1–22·3 among sites) reported in the ERSPC study.^18^ However, these differences might be because ERSPC recruited an older cohort of men (aged 55–75 years) than we did (aged 40–69 years), with a mean age of 61 years compared with 53 years in our cohort. PSA concentration increases with age, and therefore higher PSA concentrations would be expected in an older cohort. Additionally, most centres in the ERSPC study used screening intervals of 2–4 years, compared with our annual screening, and these design differences make a direct comparison between the studies challenging. The mean age in the IMPACT *BRCA1* and *BRCA2* cohort was also 54 years and 8% of men had a biopsy,^24^ which is similar to in the mismatch repair cohort and supports the idea that over-biopsy is probably not a concern in a younger cohort. The mean age of *MSH2* carriers was lower than that of *MSH2* non-carrier controls (51·9 *vs* 53·5 years) adding further weight to the difference observed in cancer incidence between carriers and non-carrier controls in this cohort.

No consensus currently exists on PSA concentration to indicate biopsy, and age-mediated PSA thresholds are being increasingly used. The PPV of biopsy using a PSA threshold of 3·0 ng/mL did not differ significantly between *MSH2* carriers and non-carrier controls (72·2% *vs* 33·3%; p=0·25) or for *MSH6* carriers and non-carrier controls (80·0% *vs* 0; p=0·14). Although the total number of cancers detected was small, these PPVs were considerably higher than those reported in the ERSPC study (24·1%), and after the baseline screening in the IMPACT *BRCA1* and *BRCA2* cohort (44%).^24^, ^30^ The PPV of PSA concentration of higher than 3·0 ng/mL for detecting cancer was also higher in *MSH2* carriers than in non-carrier controls (48·1% *vs* 14·3%; p=0·20) and in *MSH6* carriers than in non-carrier controls (80·0% *vs* 0; p=0·048). These results suggest that the use of this PSA threshold detects early-stage, clinically important disease, reflecting the higher incidence and higher grade of tumours detected in these men than in the general population. However, because the number of cancers detected was relatively small, subsequent screening rounds will be key to confirming these findings.

The incidence of prostate cancer was significantly higher in *MSH2* carriers than in non-carrier controls and in *MSH6* carriers than in non-carrier controls, adding further weight to the increased risk associated with these genes specifically; however, no specific pathogenic variant or gene region was associated with the cancer cases. The overall incidence of cancer for our study cohort was 1·9%, which is similar to the 2·4% reported in the *BRCA1* and *BRCA2* cohort of IMPACT and lower than the 4·3% of men diagnosed in the first screening round of the ERPSC.^31^ The lower incidence of prostate cancer in the IMPACT cohorts are likely explained by the younger ages of the cohorts than in ERSPC.

*MSH2* carriers were on average younger with a higher mean PSA value at diagnosis than non-carrier controls. Importantly, the incidence of clinically significant tumours (intermediate risk or high risk based on the NICE classification) was 85% (11 of 13) in the *MSH2* carriers and 75% (three of four) in the *MSH6* carriers compared with none in the two non-carrier control groups, supporting retrospective reports of a more aggressive phenotype in these groups.^6^, ^7^, ^16^, ^17^ Seven of 13 tumours diagnosed in *MSH2* carriers had Gleason 4 (grade group 3) as the dominant pattern, and three tumours were Gleason score 8 or 9 (grade groups 4–5) and so were more likely to behave aggressively with a worse prognosis. One *MSH2* carrier was found to have nodal involvement and metastatic disease at diagnosis and longer-term follow-up is required to establish whether there is a difference in metastatic events and mortality between carriers and controls. The outcome of different treatments in men with pathogenic variants in mismatch repair gene mutations and prostate cancer has not been studied (although it is under investigation as part of the GENPROS study [NCT02705846]); therefore, a minimum of 5 years' follow-up would be required to see different outcomes from treatment. Subsequent screening rounds and detection of incident cancers will be important in determining whether annual screening using a PSA threshold of 3·0 ng/mL is successful in the early detection of clinically important tumours and prevention of metastatic events.

The low incidence of prostate cancer we found, coupled with the high proportion of clinically significant disease detected at biopsy, suggests that screening men with *MSH2* and *MSH6* pathogenic variants has a low risk of overdiagnosis of indolent cancers. Because this is a baseline analysis, we had no measure of time to calculate an incidence rate ratio, but this will be included with the results of future screening rounds. No cancers were detected in either the *MLH1* carrier or non-carrier control groups and further years of follow-up are required to conclude whether or not there is an increased risk of prostate cancer associated with *MLH1*.

From a treatment perspective, knowledge of mismatch repair pathogenic variant status is increasingly important because of the evidence that mismatch repair-deficient prostate tumours can be sensitive to immune checkpoint inhibitors. The Philadelphia Prostate Cancer Consensus 2017 recommended that men with prostate cancer and a family history of Lynch syndrome should be screened for mismatch repair pathogenic variants, and men whose prostate tumour has pathogenic variants in mismatch repair genes should undergo germline testing.^32^ The NCCN guidelines support the use of the PD-1 inhibitor pembrolizumab in patients with mismatch repair-deficient, metastatic, castration-resistant, prostate cancer whose disease has progressed on at least one line of treatment.^33^ Therefore, although used predominantly in the metastatic context at present, this field is rapidly evolving and we will likely see these treatments move earlier in the treatment pathway; thus knowledge of mismatch repair status has the potential to substantially affect a patient's treatment pathway. As use of these therapies increases within prostate cancer management, establishing the risk of prostate cancer, tumour characteristics, and optimal treatments will become increasingly important.

With increasing evidence of germline pathogenic variants in mismatch repair genes predisposing to prostate cancer and aggressive disease, we hypothesise that prostate cancer screening and management guidelines will be expanded to include men with pathogenic variants in mismatch repair genes and other relevant germline variants over the coming years. Our study adds to the evidence that PSA screening identifies clinically significant prostate cancer when targeted at higher-risk groups of men. If validated in future screening rounds, there will be a strong case to adopt screening for men with pathogenic variants in mismatch repair genes into clinical guidelines. All men with pathogenic variants in mismatch repair genes or concerned about their family history of prostate cancer should discuss PSA screening with their primary care provider.

Our study had several limitations. Although the recruitment of men with these rare variants was challenging, particularly for *MSH6* carriers, we detected a significantly higher risk of prostate cancer in this cohort than in non-carrier controls. However, the number of cancers detected was relatively small and therefore further data from subsequent screening rounds are required to increase power and confirm these findings. Recruitment to the control groups was also below the initial targets, but we were able to make use of the established dataset from our *BRCA1* and *BRCA2* control group, who underwent an identical screening protocol, to enhance the numbers.

The observed mean PSA concentration at prostate cancer diagnosis in *MSH2* carriers was higher than *MSH2* non-carriers (19·7 ng/mL *vs* 5·3 ng/mL); although this difference was not significant (p=0·56). Monitoring these readings will be important in future screening rounds to determine whether or not *MSH2* carriers should undergo earlier screening or use a lower threshold for investigations than non-carriers, in view of the fact that the majority of people in this group had high-risk disease. The IMPACT protocol triggers prostate biopsy with a PSA concentration higher than 3·0 ng/mL but the outcome of biopsy at lower PSA concentrations requires further evaluation. When the protocol was initially reviewed in 2005, we were not given approval to offer biopsy at lower PSA values.

Findings from published studies of the risk of prostate cancer in Lynch syndrome have been compiled in the Prospective Lynch Syndrome Database. Most of these studies were in men of European ancestry, as indeed is the case in this IMPACT cohort (94% of those with available data were of European ancestry); therefore, generalising these findings to non-European populations will need further research. Although we had low ethnic diversity in our study, it is important to ensure that recruitment and access to trials is inclusive and reflects the diversity of the population served.

A challenge of a longitudinal study across multiple countries is in balancing the standardisation of procedures and changes in practice. In addition to the introduction of routine multiparametric MRI in the diagnostic pathway, we have seen a shift from transrectal to transperineal biopsies combined with targeted sampling of suspicious or equivocal MRI lesions. Consequently, we observed a range in the number of biopsy cores taken for diagnosis (range six to 33). We found no significant difference in the mean number of biopsy cores sampled between men with and without cancer. Fewer samples were probably taken in men for whom a targeted biopsy approach was used. This approach improves the sampling of areas with suspicious lesions, but might affect incidence of cancer. As follow-up continues over the next 5 years, future cancer diagnoses will be captured and the effect of sampling differences determined.

Without a systematic assessment of the use of MRI in men at genetically high risk of prostate cancer and its incidence in this specific subgroup, it is difficult to extrapolate general population data to this setting and further research is required. Assessing the generalisability of the results for contemporary patients, where guidelines include MRI, is also difficult. This question is being addressed in the PROFILE study (NCT02543905), in which men with pathogenic variants in genes including the mismatch repair genes will be following a screening algorithm that includes MRI. Therefore, future comparisons with the IMPACT dataset will be possible. We cannot exclude that some men might have had PSA screening before inclusion in the IMPACT study. From our participant-completed questionnaire, 30–40% of carriers and non-carriers reported undergoing PSA testing before enrolling, which might have introduced some selection bias. However, previous screening would potentially have a positive bias on the data because it would mean that only men with low PSA concentrations and those who have not undergone a prostate biopsy within the past 12 months would meet the inclusion criteria.

Not all men complied with the study protocol, and therefore cancers might have been missed either in men who refused biopsy, who had a normal MRI, or those advised locally to have a repeat PSA screening or MRI instead of a biopsy. Genetic status might affect protocol compliance, with fewer non-carriers proceeding with prostate biopsy than carriers (eg, 67% of *MSH2* carriers *vs* 43% of non-carrier controls), which might represent variation in how men are counselled, with a bias towards promoting biopsy in *MSH2* carriers. Further screening rounds with increased numbers of biopsies will enable further evaluation of this within all gene groups. If the compliance rates of the carriers and the non-carrier cohort were the same (as investigated in our sensitivity analysis), there would be no change in significance observed for *MSH2* carriers *vs* non-carrier controls. Because of the smaller number of participants in the *MSH6* cohort than in the *MSH1* and *MSH2* cohorts**,** we cannot rule out that these findings would remain significant if we had recruited larger numbers of men from these cohorts. Further screening rounds will be needed to provide additional data to support these observations. There might be some selection bias introduced with the use of local MRI in some centres, with those men with a visible lesion being put forward for biopsy. We know only four men with a negative MRI did not proceed with biopsy, so this bias is likely to be small. Because of the large number of centres and clinicians involved in the study, consistency was difficult to achieve and led to some PSA screenings being repeated on the basis of clinical discussions, rather than management being informed by a single PSA value as stated in the protocol. Because PSA screening continues for another 5 years, the clinical outcome in those men who declined prostate biopsy at this first screening round will be included in future analyses.

Finally, we need to consider that men with Lynch syndrome might have below-average life expectancy and therefore there could be a higher probability that screen-detected cancers might not otherwise have been found in a man's lifetime, and subsequently be considered to be over-detection. Further screening rounds and longer-term follow-up within the IMPACT study will enable a more complete assessment of the possible benefit and harms of screening in terms of risk of competing mortality and efficacy of early detection and treatment in these men, as well as enabling the assessment of the rates of interval cancers to inform the optimal PSA screening interval.

In summary, the first screening round of the mismatch repair cohort of IMPACT supports consideration of targeted PSA screening for prostate cancer in men with *MSH2* and *MSH6* pathogenic variants to increase the detection of prostate tumours that are highly likely to need treatment based on national and international guidelines. Using a PSA threshold of 3·0 ng/mL resulted in a low biopsy rate (6%) and a high PPV for the detection of intermediate-risk and high-risk disease in *MSH2* and *MSH6* carriers. We observed a significant difference in the incidence of prostate cancer between carriers and age-matched non-carriers. *MSH2* carriers were diagnosed at a younger age, although this was not significant, and had more clinically significant disease compared with non-carriers. Future screening rounds will determine the optimal frequency of PSA testing, the usefulness of PSA screening in *MLH1* carriers, and provide further data on the value of annual screening in *MSH2* and *MSH6* carriers.

## Data sharing

Application for access to de-identified participant data can be made to the IMPACT Study Steering Committee (impact-study@icr.ac.uk). All data access applications will be considered on their individual merits and by consensus of the Steering Committee. If applications are approved there will be no limitations on the length of time that the data are available. Data will be made available after publication of this Article. A data sharing agreement between the researcher or institutions would then be set up before release of the approved data.

## Declaration of interests

HL holds patents on intact PSA assays and is named on a patent for a statistical method to detect prostate cancer licensed to Arctic Partners and commercialised by OPKO Health, and has stock in Arctic Partners and OPKO Health and receives royalties from sales of the *4Kscore* test. RAE has received speaker honoraria from Genitourinary-American Society of Clinical Oncology, The University of Chicago, European Society for Medical Oncology (paid by Bayer and Ipsen), and The Royal Marsden NHS Foundation Trust (with support from Janssen), and is a member of the AstraZeneca UK Limited Prostate Dx Advisory Panel external expert committee. No organisation had any role in the decision to publish or in the writing of the manuscript. All other authors declare no competing interests.
