# Supplementary material for: A prospective prostate cancer screening programme for men with pathogenic variants in mismatch repair genes (IMPACT): initial results from an international prospective study
Source: Lancet Oncol. 2021 Nov;22(11):1618–31. doi: 10.1016/S1470-2045(21)00522-2 (PMC8576477; doi:10.1016/S1470-2045(21)00522-2)
Supplement: Supplementary appendix [file mmc1.pdf]

THE LANCET
Oncology

Supplementary appendix

This appendix formed part of the original submission and has been peer reviewed. We post it as supplied by the authors.

Supplement to: Bancroft EK, Page EC, Brook MN, et al. A prospective prostate cancer screening programme for men with pathogenic variants in mismatch repair genes (IMPACT): initial results from an international prospective study. *Lancet Oncol* 2021; published online Oct 19. http://dx.doi.org/10.1016/S1470-2045(21)00522-2.

The IMPACT Study Collaborators and Steering Committee – Lynch cohort

IMPACT Study Steering Committee:
PI: Prof Rosalind Eeles – Institute of Cancer Research, London, UK
Dr Elizabeth Bancroft - Royal Marsden NHS Foundation Trust, London, UK
Ms Elizabeth Page - Institute of Cancer Research, London, UK
Dr Mark Brook - Institute of Cancer Research, London, UK
Dr Zsofia Kote-Jarai - Institute of Cancer Research, London, UK
Professor Neil Aaronson – The Netherlands Cancer Institute, Amsterdam, The Netherlands
Mrs Audrey Ardern-Jones – Royal Marsden NHS Foundation Trust, London, UK
Prof Dr Chris Bangma – Erasmus Cancer Insititute, Erasmus University Medical Center, Rotterdam, The Netherlands
Dr Elena Castro – Spanish National Cancer Research Center, Madrid, Spain
Professor David Dearnaley - Institute of Cancer Research, London, UK
Dr Alex Dias - Instituto Nacional de Cancer Jose de Alencar Gomes da Silva INCA, Rio de Janeiro, Brasil
Professor Diana Eccles – University of Southampton, Southampton, UK
Professor Gareth Evans - St Mary's Hospital, Manchester, UK
Professor Jorunn Eyfjord - University of Iceland, Reykjavik, Iceland
Dr Alison Falconer – Imperial College Healthcare NHS Trust, London, UK
Professor Christopher Foster - HCA Pathology Laboratories, London, UK
Professor Henrik Grönberg - University Hospital, Umea, The Netherlands
Professor Freddie C. Hamdy - University of Oxford, Oxford, UK
Dr Óskar Þór Jóhannsson - Landspitali - National University Hospital of Iceland, Reykjavik, Iceland
Dr Vincent Khoo - Royal Marsden NHS Foundation Trust, London, UK
Professor Hans Lilja – MSKCC, New York & Lund University, Malmö, Sweden.
Professor Geoffrey Lindeman – The Walter and Eliza Hall Institute of Medical Research, Parkville Victoria, Australia.
Professor Jan Lubinski - International Hereditary Cancer Center, Szczecin, Poland
Dr Lovise Maehle - Oslo University Hospital, Oslo, Norway
Mr Alan Millner - Royal Marsden NHS Foundation Trust, London, UK
Dr Christos Mikropoulos - Royal Surrey County Hospital, Guildford
Dr Anita V. Mitra – University College London Hospitals NHS Foundation Trust
Ms Clare Moynihan - Institute of Cancer Research, London, UK
Dr Judith Offman – Guy's Hospital, London, UK
Dr Gad Rennert - CHS National Cancer Control Center, Carmel Medical Center, Haifa, UK
Dr Lucy Side – Wessex Clinical Genetics Service, Southampton, UK
Dr Mohnish Suri - Clinical Genetics Service, Nottingham University Hospitals NHS Trust, Nottingham
Dr Holly Ni Raghallaigh – The Institute of Cancer Research

Retired members of the Steering Committee:
Dr Penny Wilson - Innovate, UK
Dr Jane Melia – University of Cambridge, Cambridge, UK
Dr Gillian Mitchell - Peter MacCallum Cancer Institute, Victoria, Australia
Prof Sue Moss - Queen Mary University of London, UK
Prof Fritz Schroder - Erasmus University Medical Center, Rotterdam, The Netherlands
Prof Doug Easton - University of Cambridge, Cambridge, UK
Susan Peock - University of Cambridge, Cambridge, UK
Paul Sibley – Siemens Healthcare Diagnostics, UK
Reza Sharifi – St Georges Hospital , London, UK

Coordinating Centre, Institute of Cancer Research, London: Rosalind Eeles, Elizabeth Bancroft, Elizabeth Page, Sarah Thomas, Jenny Pope, Anthony Chamberlain, Romayne McMahon, Natalie Taylor, Kathryn Myhill, Denzil James, Matthew Hogben, Barbara Benton, Ann-Britt Jones, Jana McHugh.

Data and Safety Monitoring Committee: Stephen Duffy (Chair), Dina Patel (UK NEQAS representative), and John McGrath (BAUS representative), and past members Peter White and Richard Pocock.

IMPACT Collaborating Sites

Australia (*more than 1 affiliation)

Peter MacCallum Cancer Centre and The Royal Melbourne Hospital (including the Parkville Familial Cancer Centre), Melbourne, VIC: Geoffrey Lindeman, Paul James, Gillian Mitchell, Sue Shanley, Kate Richardson, Joanne McKinley, Lara Petelin, Morgan Murphy, Lyon Mascarenhas, Catherine Morton, Kylie Shackleton, Finlay Macrae, Phoebe Martin, Declan Murphy
Flinders Medical Centre, Bedford Park, SA: Jimmy Lam, Michael Chong
Royal Adelaide Hospital, Central Adelaide Local Health Network, Adelaide SA: Nicola Poplawski* , SA Pathology
Adelaide Medical School, University of Adelaide, Adelaide, SA: Nicola Poplawski*
Monash Health, Clayton, VIC: Marion Harris, Shona O'Connell, Clare Hunt, Courtney Smyth, Mark Frydenberg
Genetic Services of WA, Subiaco, WA: Nicholas Pachter, Sharron Townshend, Lyn Schofield
Hunter Family Cancer Service, Waratah, NSW: Allan Spigelman, Margaret Gleeson
University of Newcastle, Newcastle, NSW: Rodney Scott
Victorian Cancer Biobank, Melbourne, VIC
Pathwest (Clinical Trials Lab), WA

Israel
The Genetic Institute, The Gastroenterolgy Institute and the Urology Department, Kaplan Medical Centre, Rehovot; Rakefet Chen-Shtoyerman*, Alon Basevitch, Dan Leibovici, Ehud Melzer and Sagi Josefsberg Ben-Yehoshua
Ariel University, Ariel: Rakefet Chen-Shtoyerman*
Italy
Istituto Nazionale dei Tumori, Milan: Nicola Nicolai, Paolo Radice, Riccardo Valdagni, Tiziana Magnani, Fabiana Zollo, Mario Catanzaro, Margherita Sorrentino, Simona Gay, Marco Vitellaro.

Norway
Oslo University Hospital: Lovise Mæhle, Eli Marie Grindedal, Eldbjørg Hanslien
Akershus University Hospital: Karol Axcrona

Portugal
Portuguese Oncology Institute, Porto: Manuel Teixeira, Sofia Maia, Marta Cardoso, Ana Peixoto, Rui Henrique, Jorge Oliveira, Nuno Gonçalves, Luís Araújo, João Paulo Souto, Pedro Nogueira.

Spain
Hospital de Sant Pau, Barcelona: Teresa Ramón y Cajal, Nuria Calvo Verges, Josefina Mora, Joan Palou, Consol López, Alexandra Gisbert, Francesco Sanguedolce, Rosa Alfonso.

United Kingdom
Royal Marsden NHS Foundation Trust: Angela George, Zoe Kemp Jennifer Wiggins, , Terri McVeigh, Cathryn Moss, Lizzie Verdon, Vincent Khoo, Nicholas Van As, Alan Thompson, Chris Ogden, Christopher Woodhouse, Pardeep Kumar, Declan Cahill, Netty Kinsella, Claire McNally.
Manchester Centre for Genomic Medicine, Manchester: D Gareth Evans, Jeanette Rothwell, Fiona Lalloo, Kate Green, Karen Tricker, Barbara Bulman.
Wessex Clinical Genetics Service, Southampton: Lucy Side, Diana Eccles, Tessy Thomas, April Ruiz, Darran Ball, Oliver Jones, Catherine Mercer, Donna McBride, Philandra Costello Gillian Wise, Allison Pearce, Victoria Sands, Audrey Torokwa,
East Anglian Regional Genetics Service, Cambridge: Marc Tischkowitz, Amy Taylor, Vincent Gnanapragasam.
Oxford Regional Genetics Service, Oxford: Lisa Walker, Dorothy Halliday, Helen Purnell, Barbara Stayner, Kathryn Saunders Angela Bloss, Peter Risby, Joyce Solomons, Lara Hawkes.
South West Thames Regional Genetics Service, London: Katie Snape, Helen Hanson, Kelly Kohut, Merrie Manalo, Darshna Dudakia, Siti Ismail, Sally Goff

Peninsula Clinical Genetics Service, Exeter: Carole Brewer, Linda Park, Joanna Ireland, Alison Potter, Caroline Renton, Anne Searle, Kathryn Hill, Selina Goodman, Lynda Garcia, Gemma Devlin, Sarah Everest, Maria Nadolski, Debbie Fuller, Catherine Gray, Melanie Hutchings, Karin Gupwell, Maggie Thomas, Matilda Bradford, Sandra Cookson, Lisa Adams.
Northern Clinical Genetics Service, Newcastle: Ashraf Azzabi, Irene Jobson, Edgar Paez. Alex Henderson, Fiona Douglas,
South West Regional Genetics Service, Bristol: Alan Donaldson, Amy Watford.
South East Thames Regional Genetics Service, Guys Hospital London: Louise Izatt, Vishakha Tripathi, Michelle Weston, Kathrine Hilario, Merrie Manalo, Gabriella Pichert, Chris Jacobs, Mark Mencias, Anjana Kulkarni, Bianca De Souza, Clare Turnbull, Alice Youngs, Cecilia Compton, Adam Shaw.
North West Thames Regional Genetics Service, Harrow:
Catherine Hartigan, Cheryl Berlin, Demetra Georgiou, Nadia Preitner, Lauren Limb, Marion Bartlett, Monika Kosicka-Slawinska, Hannah Shipman, Sharon Jenkins, Natalie Ellery, Ravinder Sehra, Ilana Weintroub, Madeline Gale, Alice Callard, Anaar Sajoo, Angela Brady, Virginia Clowes, Rita Ibitoye, Bianca DeSouza.
North East Thames Regional Genetics Service, NE Thames: Munaza Ahmed, Alison Male, Jana Gurasashvili, Kate Simon, Katie Rees, Cecilia Compton, Lizzie Tidey, Laura Coulier, Camila Gabriel, Ellen Quinn, Nita Solanky.
West Midlands Regional Clinical Genetics Service, Birmingham: Kai-Ren Ong, Jonathan Hoffman, Camilla Huber, Wayne Glover, Farah Islam, Saira Ali, Lucy Burgess, Rachel Hart, Cyril Chapman, Tricia Heaton, Trevor Cole, Emma Douglas.
West of Scotland Genetics Service, Glasgow: Rosemarie Davidson, Mark Longmuir, Cathy Watt, Alexis Duncan, Nicola Bradshaw, Lesley Snadden, Jennifer Gorrie.
Leicester Royal Infirmary: Julian Barwell, Roger Kockelbergh, Shumi Mzazi, Charlotte Poile, Mandy LeButt Ayisha Sattar, Beckie Kaemba, Zahirah Sidat, Nafisa Patel, Kas Siguake, Amy Branson.
Liverpool Womens NHS Foundation Trust: Lynn Greenhalgh, Michaela Davies, Queenstone Barker Rachael O'Keefe, Anne Johnstone.
Royal Liverpool and Broadgreen Hospital NHS Trust, Liverpool: Philip Cornford, Nicola Bermingham Katy Treherne, Julie Griffiths, Pembe Yesildag
Derriford Hospital, Plymouth: Carole Brewer, Lyn Cogley, Hannah Gott, Maria Brennan, Natale Salvatore, Sue Freemantle
Urology Department, New Cross Hospital, Wolverhampton: Peter Cooke, Vanda Carter, Anna Grant, Claire Lomas, Jason Rogers
Clinical Genetics Service, Nottingham University Hospitals NHS Trust, Nottingham : Mohnish Suri, Rebecca Collier, Angela Andrew
Yorkshire regional Genetics Service, Leeds Teaching Hospitals NHS Trust, Leeds: Hannah Musgrave, Thomas Inglehearn, Julian Adlard, Nazya Azam, Jessica Maiden .

United States
NorthShore University HealthSystem, Evanston: Brian Helfand, Elena Genova, Christina Selkirk, Peter Hulick, Charles Brendler, Michael McGuire, Karen Kaul, Daniel Shevrin, Scott Weissman, Anna Newlin, Kristen Vogel, Shelly Weiss.
Huntsman Cancer Institute, University of Utah Health, Salt Lake City, Utah: Saundra Buys, David Goldgar, Karen O'Toole, Tom Conner, Vickie Venne, Robert Stephenson, Christopher Dechet.
University of Michigan: Kathy Cooney, Elena Stoffel, Linda Okoth
Dana Farber Cancer Institute, Boston: Sapna Syngal, Chinedu Ukaegbu, Caroline Clark, Brigette Rankin, Maggie Ruderman, Matthew Yurgelun, Ramona Lim, Margaret Klehm, Leah Biller, Sigurdis Haraldsdottir, Kimberley Perez, Quoc-Dien Trinh.

We would like to thank all those who have been involved with IMPACT, contributing to both our BRCA and Lynch cohorts.

Supplementary Table 1 - Study population and recruitment countries and centres, grouped by genetic status*

Site, Country (Principal Investigator)	*MLH1* Carriers	*MLH1 Non* Carriers	*MSH2* Carriers	*MSH2 Non* Carriers	*MSH6* Carriers	*MSH6 Non* Carriers	Total
Birmingham Womens Hospital, Birmingham, UK (Kai Ren Ong)	33	29	44	28	20	19	173
Norwegian Radium Hospital, Oslo, Norway (Lovise Mæhle)	14	3	35	0	19	4	75
St Marys Hospital Manchester, Manchester, UK (D. Gareth Evans)	26	2	28	2	14	0	72
Northwick Park Hospital, London, UK (Angela Brady)	17	5	8	4	5	2	41
St Georges Hospital, London, UK (Katie Snape)	14	1	13	0	11	0	39
Peter MacCallum Cancer Centre, VIC, Australia (Geoffrey Lindeman)	5	1	17	7	5	2	37
Dana Farber Cancer Center, Boston, USA (Sapna Syngal)	14	0	11	0	6	1	32
Princess Anne Hospital, Southampton, UK (Lucy Side)	10	0	14	1	5	0	30
University Hospitals of Leicester. Leicester, UK (Julian Barwell)	5	4	10	3	5	1	28
Portuguese Oncology Institute, Porto, Portugal (Manuel Teixeira)	3	8	9	5	1	1	27
Guys Hospital, London, UK (Louise Izatt)	9	0	12	1	3	0	25
Nottingham University Hospital, Nottingham, UK (Mohnish Suri)	2	2	7	2	8	4	25
Royal Melbourne Hospital, VIC, Australia (Paul James)	6	1	4	3	6	2	22
Repatriation General, SA, Australia (Nicola Poplawski)	6	4	2	3	1	2	18
Kaplan Medial Center, Rehovot, Israel (Alon Basevitch)	1	1	5	6	3	0	16
Great Ormond Street Hospital, London, UK (Munaza Ahmed)	3	1	10	0	1	0	15
Leeds Teaching Hospital, Leeds, UK (Julian Adlard)	4	0	9	0	2	0	15
IRCCS Istituto Nazionale dei Tumori, Milan, Italy (Nicola Nicolai)	6	0	7	0	1	0	14
Royal Devon and Exeter, Exeter, UK (Carole Brewer)	3	0	7	0	3	0	13
Liverpool Womens Hospital, Liverpool, UK (Lynn Greenhalgh)	1	0	8	4	0	0	13
King Edward Hospital, WA, Australia (Nicholas Pachter)	3	0	8	0	1	0	12
Royal Marsden Hospital, London, UK (Rosalind Eeles)	4	0	5	0	0	2	11

Supplementary Table 1 cont…							
**Site, Country (Principal Investigator)**	***MLH1* Carriers**	***MLH1* Non Carriers**	***MSH2* Carriers**	***MSH2* Non Carriers**	***MSH6* Carriers**	***MSH6* Non Carriers**	**Total**
**Hunter Family Cancer Service, NSW, Australia (Allan Spigelmam)**	1	1	4	2	1	1	10
**Freeman Hospital, Newcastle, UK (Ashraf Azzabi)**	5	1	2	0	1	0	9
**NorthShore University HealthSystem, Evanston, USA (Brian Helfand)**	0	1	5	2	0	0	8
**Churchill Hospital Oxford, UK (Dorothy Halliday)**	1	0	5	0	1	0	7
**University of Utah, Salt Lake City, USA (Saundra Buys)**	3	0	1	1	1	1	7
**St Michaels Hospital, Bristol, UK (Alan Donaldson)**	0	0	3	0	3	0	6
**University of Michigan Comprehensive Cancer Center, Michigan, USA (Kathy Cooney)**	1	0	4	0	1	0	6
**Hospital de Sant Pau, Barcelona, Spain (Teresa Ramón y Cajal)**	3	0	1	0	2	0	6
**Monash Health, VIC, Australia (Marion Harris)**	0	0	0	2	2	1	5
**Derriford Hospital, Plymouth, UK (Carole Brewer)**	0	0	4	0	0	0	4
**Queen Elizabeth University Hospital, Glasgow, UK (Rosemarie Davidson)**	0	0	2	0	2	0	4
**Addenbrookes Hospital, Cambridge, UK (Marc Tischkowitz)**	1	0	1	0	1	0	3
**Grand Total**	**204**	**65**	**305**	**76**	**135**	**43**	**828**

*Does not include additional 134 *BRCA1/2* non-carriers.
